# Gestational age determination in pregnancies conceived via assisted reproductive technology

**DOI:** 10.1002/uog.70219

**Published:** 2026-04-30

**Authors:** M. Gjerdevik, H. I. Hanevik, S. E. Håberg, H. K. Gjessing

**Affiliations:** ^1^ Department of Computer Science, Electrical Engineering and Mathematical Sciences Western Norway University of Applied Sciences Bergen Norway; ^2^ Centre for Fertility and Health Norwegian Institute of Public Health Oslo Norway; ^3^ Fertility Department Telemark Hospital Trust Skien Norway; ^4^ Department of Global Public Health and Primary Care University of Bergen Bergen Norway

**Keywords:** assisted reproductive technology (ART), estimated date of delivery, fresh and frozen embryo transfer, gestational age, pregnancy duration, time‐to‐event analysis, ultrasound

## Abstract

**Objectives:**

To compare gestational age (GA) dating models for pregnancies conceived via assisted reproductive technology (ART), assess whether 14 or 15 days more accurately reflects the median follicular‐phase duration in ART pregnancies and evaluate whether the gestational timelines of ART pregnancies are comparable with that of spontaneously conceived pregnancies.

**Methods:**

We employed population data from the Medical Birth Registry of Norway (2015–2021), including 163 544 children conceived spontaneously, 2067 conceived via fresh embryo transfer (ET) and 2080 conceived via frozen ET. Among ART pregnancies, we compared two GA dating methods: a population‐based ultrasound model (GA_US_) and an ART‐based formula based on the known oocyte fertilization date. Statistical agreement was evaluated by calculating individual pairwise differences in GA estimates. Bias and precision in the estimated date of delivery (EDD) were assessed by constructing cumulative birth distribution (Kaplan–Meier) curves for fresh and frozen ET pregnancies separately, employing time‐to‐event analysis to account for nonspontaneous births.

**Results:**

The absolute individual GA differences between ultrasound and ART‐based dating, considering a 14‐day median follicular‐phase duration, were ≤ 1 day for 969/2067 (46.9%) fresh ET pregnancies and for 874/2080 (42.0%) frozen ET pregnancies. An approximate systematic difference of 1 day was observed, which we corrected by employing a 15‐day median follicular‐phase duration in the ART‐based formula (GA_ART,15_). There was a median pairwise difference between GA_US_ and GA_ART,15_ of −0.4 (95% CI, −0.5 to −0.3) days in fresh ET pregnancies and −0.2 (95% CI, −0.4 to −0.1) days in frozen ET pregnancies. Both models demonstrated equivalent precision in the EDD. Measured using the ultrasound model, frozen ET pregnancies had a median pregnancy duration of 286.1 (95% CI, 285.5–286.7) days, which was 3.2 (95% CI, 2.4–3.9) days longer than that in fresh ET pregnancies, and 2.6 (95% CI, 2.0–3.2) days longer than that in spontaneously conceived pregnancies.

**Conclusions:**

We found close statistical agreement between ultrasound and ART‐based dating models for both GA and EDD. However, 15 (not 14) days of follicular‐phase duration should be utilized in the ART‐based formula to avoid systematic bias in ART‐based GA estimates. Fresh and frozen ET pregnancies had different birth distributions and median pregnancy durations, underscoring that they are not comparable with spontaneously conceived pregnancies and they should be evaluated separately. © 2026 The Author(s). *Ultrasound in Obstetrics & Gynecology* published by John Wiley & Sons Ltd on behalf of International Society of Ultrasound in Obstetrics and Gynecology.

## Abstract 2

Linked article: There is a comment on this article by Cavoretto et al. Click here to view the Editorial.

## INTRODUCTION

In modern obstetric care, fetal ultrasound biometry in the late first or early second trimester of pregnancy is widely used to calculate gestational age (GA) and the estimated date of delivery (EDD). For pregnancies conceived via assisted reproductive technology (ART), the date of oocyte fertilization (usually the same as the date of oocyte retrieval) or the date of embryo transfer (ET) is commonly used to calculate GA and EDD.

Several dating charts have been developed by estimating the ET‐derived GA of ART‐conceived pregnancies using fetal ultrasound biometry, aiming to utilize these charts in spontaneously conceived pregnancies^1^, ^2^, ^3^, ^4^, ^5^, ^6^, ^7^, ^8^, ^9^. However, this approach overlooks a possible delay between ovulation and fertilization in spontaneously conceived pregnancies, as well as *in‐vitro* and *in‐utero* differences in the speed of early embryonic development^10^. Furthermore, ART pregnancies are a selected subpopulation. Thus, elevated risks of perinatal complications^11^ and preterm birth^12^, ^13^ may distort the birth distribution of ART pregnancies compared with that of spontaneously conceived pregnancies, potentially reducing the usefulness of ART‐derived dating charts for the latter.

Since the GA scale, by historical convention, starts on the first day of the last menstrual period (LMP), all dating methods must be calibrated to match the start of the LMP^14^. GA calculations based on ART customarily add 14 days to the date of oocyte fertilization to account for the median follicular‐phase duration, although it remains uncertain whether this assumption provides correct estimates for ART pregnancies^1^, ^2^, ^15^, ^16^.

We aimed to investigate individual agreement in GA calculations between a standardized population‐based ultrasound dating model and the ART‐based formula in both fresh and frozen ET pregnancies (i.e. to calculate the pairwise difference between GAs calculated by the two different methods in the same pregnancy). In particular, we explored the validity of using 14 days *vs* 15 days to account for the median follicular‐phase duration in ART‐based dating. Employing population data from the Medical Birth Registry of Norway (MBRN) (2015–2021)^17^, ^18^, we constructed cumulative birth‐distribution curves and used censored time‐to‐event analysis to compensate for the increasing proportions of induced births and elective Cesarean sections^19^. Within this time‐to‐event framework, we evaluated the bias and precision in EDD and compared the median duration of pregnancies conceived spontaneously *vs* those conceived using ART.

## METHODS

### Data collection

In Norway, prenatal care for pregnant women is provided as part of the public healthcare system. We established our study cohort from the MBRN, in which information on all births in Norway has been registered since 1967^17^, ^18^. The mandatory birth notification form includes information about maternal health before and during pregnancy, complications occurring during pregnancy and birth and details on ART treatment.

In 2015, the MBRN started recording the specific dating method used for each pregnancy. The number of recordings was limited initially but has increased in recent years. To obtain consistent ultrasound dating for all pregnancies, we restricted our cohort to pregnancies known to be dated specifically using the prediction model ‘eSnurra’ (defined below), thus avoiding biases introduced with other dating models. Thus, we included in our cohort children born at 22 weeks' gestation or later between January 2015 and December 2021, with their pregnancy recorded specifically in the MBRN as having been dated by eSnurra.

This study was approved by the Regional Committee for Medical and Health Research Ethics for South/East Norway (#2014/404).

### Dating by last menstrual period

Before the advent of ultrasound examination, GA was based on the LMP, considering the first day of the LMP as day 0. Although supplanted almost universally by ultrasound and ART‐based dating, the first day of the LMP remains the definition of day 0 of a pregnancy. Even if the LMP is unknown for a given pregnancy, all pregnancy dating methods, whether using ultrasound or ART‐based dating, must align with this starting value. Starting from the LMP, the median GA at birth (i.e. median duration of pregnancy) has been found to be approximately 283 days when using birth records with certain and regular LMP dates, in both Norwegian and other populations^20^, ^21^, ^22^. Gestational day 283 thus defines the EDD from the first day of the LMP.

### Ultrasound dating

From an international perspective, several models for pregnancy dating using ultrasound have been published. However, traditional models are often sample‐based, and few models have been actually developed, calibrated and tested in a population setting, therefore potentially exposing them to systematic biases in their calculations of GA and EDD^23^, ^24^, ^25^. Such biases will affect any comparison between ultrasound and ART‐based dating.

Since approximately 1984 and until recently, ultrasound dating scans in Norway have routinely been performed at around week 18 of pregnancy. In Norway, the predominant method for calculating GA and EDD since 2007 has been ‘eSnurra’ (https://www.esnurra.com), a prediction model that employs a standardized, population‐based approach for ultrasound dating based on fetal size parameters such as biparietal diameter (BPD), femur length and crown–rump length (CRL)^14^, ^22^.

Built into eSnurra is a calibration assuming a median GA at birth of 283 days. This calibration ensures that the start of the eSnurra GA scale is properly aligned with the start of the LMP GA scale. Thus, it ensures that there is no systematic difference between GA calculations based on the LMP and those based on eSnurra; the median difference will, by calibration, be close to zero, and the ultrasound‐based EDD will fall on day 283^14^, ^22^. Consequently, we chose GA calculated using ultrasound by eSnurra (GA_US_) to compare with ART‐based GA. The MBRN records the estimated value of GA_US_ at birth. From this, we can calculate the corresponding GA_US_ at any date prior to birth (see Appendix S1 for details).

### Dating of ART pregnancies by transfer date

In the MBRN, the GA at birth for fresh ET pregnancies is calculated using the formula: GA_ART,14_ at birth = 14 days + (days from oocyte fertilization to ET) + (days from ET to birth).

As with GA_US_, the MBRN‐recorded GA_ART,14_ at birth can be used to calculate GA_ART,14_ at any date prior to birth. The GA_ART,14_ calculation for frozen ET pregnancies is equivalent to that of fresh ET, but the second term ‘days from oocyte fertilization to ET’ is replaced by ‘days from oocyte fertilization to embryo cryopreservation (EC)’. The 14 days in GA_ART,14_ are assumed to be a reasonable approximation of the median follicular‐phase duration and intend to align the ART‐based dating scale with the conventional LMP scale. However, the rationale for using 14 days for this approximation in ART‐based dating is weak. We conducted extensive analyses, described in more detail below, to assess critically the appropriateness of this value. As a result, we compared the duration of 14 days to an alternative duration of 15 days (using the corresponding formula GA_ART,15_), aiming to minimize potential systematic bias in the timescale calibration. The formulae GA_ART,14_ and GA_ART,15_ both include a term for the number of days from oocyte fertilization to ET (or EC). Thus, the formulae assume that the effective ‘start of the gestational clock’ is the day of fertilization rather than the day of transfer. We evaluated carefully how well this assumption fitted our data.

### Statistical analysis

To investigate the agreement between ultrasound‐ and ART‐based GA calculations, we calculated the pairwise difference in GA derived using these two methods within each ART‐conceived pregnancy (GA_US_ − GA_ART,14_). Data on fresh and frozen ET pregnancies were analyzed separately. The distribution of pairwise differences was assessed using histograms, and we calculated the median of the pairwise difference and a frequency table for the difference (in days). Note that the pairwise difference GA_US_ − GA_ART,14_ remains the same throughout pregnancy and is not affected by whether or not the onset of birth occurs spontaneously. For comparison, we also calculated the median of the pairwise difference GA_US_ − GA_ART,15_.

Furthermore, we wanted to compare median ART pregnancy durations when measured both by ultrasound and ART‐based dating, as well as the birth distributions of ART pregnancies when using either of the two dating methods. In addition, using ultrasound dating, we wanted to compare the median pregnancy duration of ART pregnancies with that of spontaneously conceived pregnancies (the latter expected to be around 283 days by calibration), and to compare their birth distributions. Comparisons should thus investigate both systematic differences in median pregnancy duration (bias) as well as the shape of the birth distribution around the median duration (precision).

A key challenge when evaluating birth distributions is the substantial number of births with nonspontaneous onset in contemporary pregnancy care. For instance, a considerable proportion of pregnancies are induced or delivered via elective Cesarean section prior to the EDD. This shifts the birth distribution to lower GAs than what would be seen in a distribution of spontaneous births. If the clinical strategy for inductions and Cesarean sections differs between spontaneously conceived and ART pregnancies, comparing the birth distributions of the groups may be misleading, as it would reflect differences in clinical care rather than underlying biological differences. This problem is not solved readily by excluding nonspontaneous births, as this strategy also causes biased results and unnecessarily omits data. An effective approach to handling nonspontaneous births is time‐to‐event analysis, also known as survival analysis^26^. In our framework, spontaneous births were treated as events, and nonspontaneous births were assumed to be ‘right‐censored’^21^, ^22^. The time‐to‐event analyses provide Kaplan–Meier estimates of the 'survival function' *S*(*t*), which is the proportion of pregnancies still ongoing at time *t*, in which *t* is the calculated GA. Equivalently, the cumulative birth distribution can be calculated as *F*(*t*) = 1 − *S*(*t*), in which *F*(*t*) is the proportion of pregnant women who have delivered at or before time *t*. We calculated the median GA at birth from the survival functions for spontaneously conceived pregnancies, as well as for fresh and frozen ET pregnancies, and compared their values. Statistical differences in median GA at birth were assessed using 95% CIs, obtained by parametric Monte Carlo simulations under the standard asymptotic normality assumption of the log‐transformed survival values around the median. In the ART groups, GA was calculated by ultrasound (GA_US_) as well as ART‐based dating (using both GA_ART,14_ and GA_ART,15_ for comparison). Details are provided in Appendix S2.

ART‐based GA estimates incorporate the number of days from oocyte fertilization to ET (or days from oocyte fertilization to EC). Implicitly, this assumes that ‘the clock starts ticking’ on the day of oocyte fertilization (presumed to be the day of oocyte retrieval) and not the day of transfer. We tested this assumption in two ways. First, we examined the effect of the number of days between oocyte fertilization and ET (or EC) on the ultrasound‐based GA on the day of transfer (i.e. for each of the day‐2, day‐3 and day‐5 ET (or EC) groups, we computed the median GA_US_ on the day of transfer to see if GA increased correspondingly). Second, we examined the effect of the number of days between oocyte fertilization and ET (or EC) on the median duration from the day of transfer to birth. The median duration from the day of transfer to birth was calculated using Kaplan–Meier estimates, treating nonspontaneous births as censored. Analyses were performed separately for fresh and frozen ET pregnancies.

We performed various sensitivity analyses to verify methodology and data selection. In particular, we investigated the potential selection bias caused by excluding births that were not dated by the population‐based ultrasound model (eSnurra) and examined the effect of different methods of ART (intracytoplasmic sperm injection (ICSI) *vs in‐vitro* fertilization (IVF)). Several covariates could have confounded our results. For example, advanced maternal age increases the likelihood of ART treatment and also affects the duration of pregnancy. We therefore performed censored quantile regression analyses, adjusting for maternal age at birth, parity and fetal sex. We also compared the cumulative GA distributions estimated by the Kaplan–Meier method with cumulative GA curves derived when excluding all nonspontaneous births, as well as with GA curves that treated nonspontaneous births as spontaneous (i.e. as events without censoring). As a fourth alternative, we considered a competing‐risks strategy. A frequent medical indication for iatrogenic delivery is pre‐eclampsia, and a commonly proposed strategy to investigate the relationship between risk factors, pre‐eclampsia and GA at birth is based on treating pre‐eclamptic births as a ‘competing risk’^27^. While a more detailed analysis of pre‐eclamptic pregnancies might provide additional insights, we restricted our competing‐risks modeling to a check of how much the competing‐risk assumption for pre‐eclamptic births would impact on our cumulative GA curves by calculating cause‐specific cumulative‐incidence curves^26^.

All analyses were performed using R (version 4.2.3; R Foundation for Statistical Computing, Vienna, Austria). The survival function was constructed through the *survival* package (version 3.5‐5).

## RESULTS

Between January 2015 and December 2021, a total of 179 810 children were born at 22 weeks' gestation or later, with their pregnancy recorded in the MBRN as having been dated by eSnurra specifically. We excluded 4580 children from multiple births, 617 pregnancies that ended in stillbirth or perinatal death and 6729 children diagnosed with congenital malformations. We also excluded three children with GA_US_ at birth > 310 days. Moreover, 190 children conceived via ART were excluded owing to missing or ambiguous information. Our final cohort included 163 544 spontaneously conceived children, 2067 conceived via ART involving fresh ET and 2080 conceived via ART involving frozen ET.

**Figure 1:**
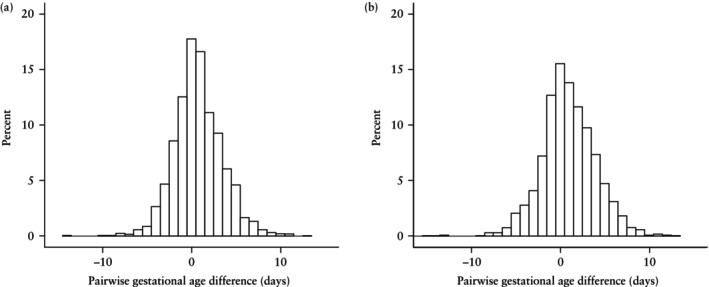
Histograms showing distribution of pairwise gestational age (GA) differences in days between the population‐based ultrasound dating model (GA_US_) and the assisted reproductive technology (ART)‐based dating model with an assumed median follicular‐phase duration of 14 days (GA_ART,14_) in: (a) fresh embryo transfer pregnancies (*n* = 2067) and (b) frozen embryo transfer pregnancies (*n* = 2080). Each pairwise difference was calculated by GA_US_ − GA_ART,14_.

Figure 1 displays the distribution of individual pairwise GA_US_ − GA_ART,14_ differences in histograms. For fresh ET pregnancies (Figure 1a), the median pairwise difference between GA_US_ and GA_ART,14_ was 0.6 (95% CI, 0.5–0.7) days. The quartiles were relatively symmetrically distributed about the median, with the 1^st^ quartile −1.5 days from the median and the 3^rd^ quartile +1.8 days from the median. The absolute GA_US_ − GA_ART,14_ difference was ≤ 1 day for 969/2067 (46.9%) pregnancies. Furthermore, 407 (19.7%) pregnancies had an absolute difference of 2 days, 288 (13.9%) had a difference of 3 days, 180 (8.7%) had a difference of 4 days and 223 (10.8%) had a difference of ≥ 5 days.

For frozen ET pregnancies (Figure 1b), the median pairwise difference between GA_US_ and GA_ART,14_ was 0.8 (95% CI, 0.6–0.9) days. The 1^st^ quartile was −1.7 days from the median and the 3^rd^ quartile was +2.1 days from the median. The absolute GA_US_ − GA_ART,14_ difference was ≤ 1 day for 874/2080 (42.0%) pregnancies, 2 days for 392 (18.8%) pregnancies, 3 days for 288 (13.8%) pregnancies, 4 days for 211 (10.1%) pregnancies and ≥ 5 days for 315 (15.1%) pregnancies.

Although the overall median GA differences between ultrasound and ART‐based dating approaches were small, these median differences were closer to zero when the median follicular‐phase duration of the ART‐based model was calibrated to be 15 days instead of 14 days. The median pairwise difference between GA_US_ and GA_ART,15_ was −0.4 (95% CI, −0.5 to −0.3) days in fresh ET pregnancies and −0.2 (95% CI, −0.4 to −0.1) days in frozen ET pregnancies. Replacing GA_ART,14_ with GA_ART,15_ also balanced the proportion of preterm births between ultrasound and ART‐based dating. The results of this recalibration are shown in Appendix S3. As shown in Figure S1, we also compared the distribution of pairwise GA_US_ − GA_ART,15_ differences with the distribution of pairwise GA differences between ultrasound and LMP‐based dating in spontaneously conceived pregnancies in our cohort, and the distribution of GA differences between BPD‐ and CRL‐based dating as reported by Gjessing *et al*.^22^.

The nonspontaneous births in our data included induced births and elective Cesarean sections, in addition to a small number of emergency Cesarean sections performed prior to the onset of labor. The observed proportions of nonspontaneous births were 30.7% (50 165/163 544) among spontaneously conceived pregnancies, 35.5% (734/2067) among fresh ET pregnancies and 44.1% (918/2080) among frozen ET pregnancies, underscoring the need for time‐to‐event analysis (Appendix S4, Figure S2).

**Figure 2:**
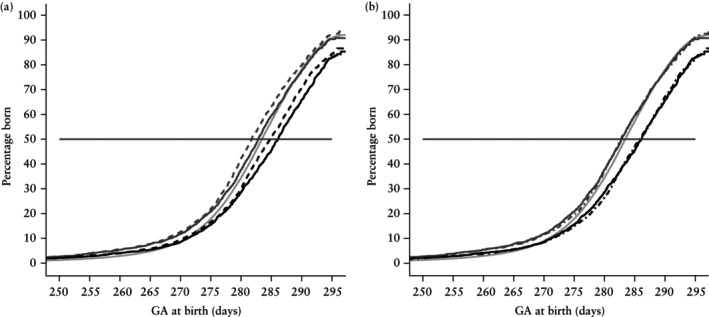
Cumulative birth distribution (Kaplan–Meier) curves comparing gestational age (GA) at birth determined using the population‐based ultrasound dating model (GA_US_) (in spontaneously conceived pregnancies (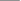), fresh embryo transfer (ET) pregnancies (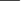) and frozen ET pregnancies (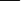)) with the assisted reproductive technology (ART)‐based dating formula using a median follicular‐phase duration of: (a) 14 days (GA_ART,14_) (in fresh ET (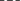) and frozen ET (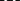) pregnancies) and (b) 15 days (GA_ART,15_) (in fresh ET (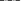) and frozen ET (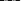) pregnancies). A time‐to‐event approach was employed, treating non‐spontaneous births as censored observations. For each curve, both the event and censoring times were recorded on the same timescale (derived from either the ultrasound or ART‐based formula).

Figure 2 presents the cumulative birth distributions for spontaneously conceived pregnancies, fresh ET pregnancies and frozen ET pregnancies, calculated according to GA_US_. In Figure 2a, the cumulative birth distributions of ART‐dated pregnancies using a follicular‐phase duration of 14 days (GA_ART,14_) are also shown. For both fresh and frozen ET pregnancies, a systematic bias of approximately one day in GA at birth was observed between GA_US_ and GA_ART,14_. Hence, we added an additional day to the ART‐determined GA, corresponding to a median follicular‐phase duration of 15 days (GA_ART,15_), as displayed in Figure 2b. For both fresh and frozen ET pregnancies, the birth distribution curves according to GA_US_ and GA_ART,15_ were approximately overlapping, demonstrating that the two methods have equivalent precision for the EDD.

Spontaneously conceived pregnancies exhibited a median GA_US_ at birth of 283.4 (95% CI, 283.4–283.5) days, which is only 0.4 days longer than the eSnurra‐calibrated 283 days. Fresh ET pregnancies showed a median GA_US_ at birth of 282.8 (95% CI, 282.4–283.4) days and frozen ET pregnancies had a median GA_US_ at birth of 286.1 (95% CI, 285.5–286.7) days. In comparison, the median GA_ART,15_ at birth was 282.7 (95% CI, 282.3–283.3) days for fresh ET pregnancies and 285.8 (95% CI, 285.1–286.5) days for frozen ET pregnancies.

The difference in the median GA_US_ at birth between fresh ET pregnancies and spontaneously conceived pregnancies was −0.6 (95% CI, −1.1 to −0.1) days. Measured by GA_US_, fetuses conceived via frozen ET were born 2.6 (95% CI, 2.0–3.2) days later than those spontaneously conceived and 3.2 (95% CI, 2.4–3.9) days later than those conceived via fresh ET. Similarly, the difference in median GA_ART,15_ at birth between frozen and fresh ET pregnancies was 3.1 (95% CI, 2.2–3.9) days. The birth distributions for both fresh and frozen ET pregnancies were clearly wider than the birth distribution for spontaneously conceived pregnancies.

**Figure 3:**
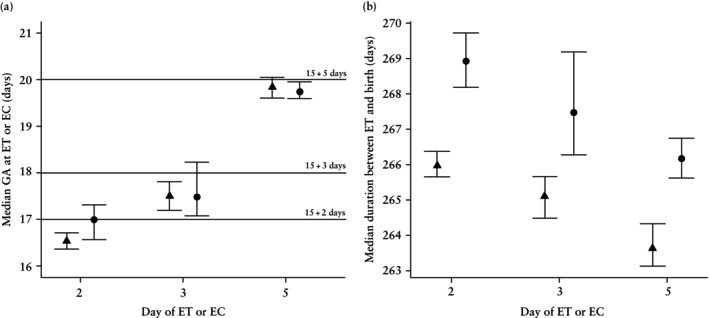
(a) Median (95% CI) gestational age (GA) of embryos on day of embryo transfer (ET) in fresh ET pregnancies (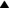) and on day of embryo cryopreservation (EC) in frozen ET pregnancies (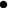) estimated using a population‐based ultrasound dating model (GA_US_), stratified by day of ET or EC after oocyte fertilization. In frozen ET pregnancies, the majority of embryos were cryopreserved on day 5 (1630/2080 embryos), resulting in wider confidence intervals for day‐2 (245 embryos) and day‐3 (117 embryos) EC. In fresh ET pregnancies, 922/2067 embryos were transferred on day 2, 500 on day 3 and 569 on day 5. (b) Kaplan–Meier estimates of the median (95% CI) number of days between ET and birth in fresh ET (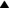) and frozen ET (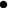) pregnancies, respectively, stratified by day of ET or EC. The remaining GA was calculated based on the extended dataset comprising 8945 fresh ET pregnancies and 4795 frozen ET pregnancies from the Medical Birth Registry of Norway (2015–2021). Larger sample sizes were available since these analyses did not involve GA_US_ (i.e. were not restricted to eSnurra samples).

Figure 3a shows the median estimated GA_US_ on the day of ET for fresh ET pregnancies, or the day of EC for frozen ET pregnancies, for the day‐2, day‐3 and day‐5 ET or EC groups (see Appendix S5, Table S1 for the group‐specific sample sizes). The median GA_US_ increased with prolonged *in‐vitro* embryo culture, demonstrating an approximate 3‐day increase between the day‐2 and day‐5 transfer groups, thus reflecting accurately the difference in the number of embryo culture days. Notably, our estimates aligned better with an assumed 15‐day rather than 14‐day follicular phase, as indicated by the horizontal lines. The similar GA estimates for the day of ET or EC for fresh and frozen ET pregnancies, respectively, suggest that there are no systematic differences in fetal size at their routine week‐18 ultrasound scans.

Figure 3b displays the Kaplan–Meier estimates of the median number of days between ET and birth. These calculations were based on an extended dataset of 8945 children conceived via fresh ET and 4795 conceived via frozen ET reported in the MBRN between 2015 and 2021 (Appendix S5, Table S2). The number of days between ET and birth decreased with prolonged *in‐vitro* embryo culture, thus accurately reflecting the number of days between oocyte fertilization and ET or EC. The estimates also show a systematic approximate 3‐day difference in duration between ET and birth between fresh and frozen ET pregnancies, similar to the difference observed in Figure 2.

The results of our sensitivity analyses are shown in Appendix S6. Our findings were consistent when applied to the extended data of all available ART samples not restricted to eSnurra samples (Figure S3), and the results were also largely unaffected by whether the fertilization method was ICSI or IVF (Figure S4). The estimates of the median GA at birth were similar after adjusting for maternal age at birth, parity and fetal sex (Figure S5). However, an extended investigation of potential confounding variables is beyond the scope of this paper. The median GA at birth was greatly underestimated if not properly accounting for nonspontaneous births, highlighting the need for time‐to‐event analysis to assess the distributions of births (Figure S6). Treating pre‐eclamptic pregnancies as a ‘competing risk’, and thus calculating a cause‐specific cumulative GA incidence curve for the remaining pregnancies, did not affect our overall findings (Figure S6).

## DISCUSSION

The commonly used ART‐based dating calibration with a 14‐day follicular‐phase duration is intended to align the start of the ART‐based GA scale with those of the LMP‐ and ultrasound‐based scales, but it might be suboptimal. Since protocols for clinical pregnancy management are developed primarily for spontaneously conceived pregnancies, even a 1‐day systematic shift in GA will impact on the management of ART pregnancies at the population level. Our GA_US_ model is specifically designed to align correctly with the LMP scale, matching day 0 of pregnancy and the median GA at birth of 283 days^22^. We observed a median pairwise difference of 0.5–1 day between GA_US_ and GA_ART,14_ for both fresh and frozen ET pregnancies. Even more pronounced, we found an approximate systematic 1‐day difference in median GA at birth between GA_US_ and GA_ART,14_. Consequently, we recommend correcting this bias by using 15 days instead of 14 days as the follicular‐phase duration in ART‐based dating.

After correcting for this 1‐day systematic shift, there was a close agreement between ultrasound‐based and ART‐based GA calculations; the pairwise differences had a narrow symmetric distribution around zero. Similarly, the cumulative birth distributions of GA_US_ and GA_ART,15_ were nearly identical for both fresh and frozen ET pregnancies, showing that ultrasound and ART‐based dating predict the time of birth equally precisely. Thus, one cannot statistically determine whether ultrasound or ART‐based dating predicts GA and EDD more accurately.

The high consistency between ultrasound and ART‐based dating for ART‐conceived pregnancies facilitates unbiased comparisons between ART and spontaneously conceived pregnancies using ultrasound dating. Fresh ET pregnancies delivered, in median, > 0.5 days before spontaneously conceived pregnancies, and frozen ET pregnancies delivered > 2.5 days after pregnancies that were spontaneously conceived. Hence, we observed an approximate difference of 3 days in the median GA at birth between fresh and frozen ET pregnancies, which was the case when using both the ultrasound and the ART‐based formulae.

This remarkable consistency between the two dating models strongly suggests that the apparent delay in birth of frozen ET pregnancies pertains to the timing of the onset of birth rather than to an early ‘hibernation’ period. Since the number of *in*‐*vitro* embryo culture days was directly reflected in the total pregnancy duration, our study strongly suggests that ‘the clock starts ticking’ at the time of oocyte fertilization and not at the time of ET. Furthermore, the systematic differences in GA at birth observed between fresh and frozen ET pregnancies were not present in our data at the routine 18‐week ultrasound examination, implying that the observed delay in birth for frozen ET pregnancies is unrelated to fetal growth in early pregnancy, and that additional factors contribute to the timing of birth in ART pregnancies. Fresh ET pregnancies had a median GA at birth similar to that of those that were spontaneously conceived, thus close to an EDD of day 283. However, the approximate delay of 3 days in the median GA at birth for frozen ET pregnancies makes an EDD of day 283 less optimal in these cases.

Compared with fresh ET pregnancies, frozen ET pregnancies are associated with increased fetal growth and birth weight^28^, a lower incidence of pregnancy‐related complications^29^ and a lower uterine artery pulsatility index^30^. Together with our findings, this underscores the need for separate risk assessments for fresh *vs* frozen ET pregnancies at various GAs. Although more studies are needed to identify potential confounders and determine the causes underlying these observed differences, it is evident that ART pregnancies are different from those conceived spontaneously, particularly in terms of duration of pregnancy. Consequently, dating models based on ART pregnancies may not be optimal for dating spontaneously conceived pregnancies.

Close agreement between ultrasound‐ and ART‐based GA estimates has been observed in several other studies^1^, ^2^, ^31^, ^32^, ^33^. Källén *et al*.^6^ reported a small underestimation of GA based on ultrasound compared with ET‐based dating. However, the differences were smaller than those observed between LMP and ultrasound dating, which aligns with our findings. The recent study of Knight *et al*.^7^ found a slight systematic overestimation of several CRL‐based ultrasound charts compared with dating based on the known oocyte fertilization date in IVF pregnancies. However, it is unclear whether either of the ultrasound‐ or ART‐based GA calculations was calibrated correctly against the LMP.

Our study underscores that both ultrasound‐ and ART‐based GA calculations can suffer from a lack of calibration. Ultrasound dating can be unbiased if properly corrected^34^, and ART‐based dating can be calibrated using 15 days rather than 14 days. When these dating methods were properly aligned by calibration, we found no difference in the precision of the EDD. While ART‐based dating has the benefit of using the known oocyte fertilization date, ultrasound dating has the benefit of aligning automatically the dating of ART pregnancies with that of spontaneously conceived pregnancies.

A limitation of our study is that we did not evaluate the dating methods against other clinical endpoints. Conceivably, small but clinically important subgroups such as fetuses with congenital anomalies or pregnancies with atypical placentation may behave differently and may not be visible in a model evaluation at the population level.

This study employed comprehensive population data collected since 2015, and the recency of the data is a strength owing to rapid advances in ART procedures over recent decades. However, we did not have direct access to ultrasound measurements of fetal biometry. Thus, subtle variations in fetal growth may have gone undetected, since our study depended on ultrasound‐based GA, as reported in days to the MBRN. To facilitate comparisons of groups with different and sizeable proportions of nonspontaneous births, we used a time‐to‐event approach and showed that alternative strategies would cause substantial bias. The underlying assumption in a time‐to‐event analysis is that pregnancies with nonspontaneous births would have followed the same birth distribution as those with spontaneous births if they had not been induced. While this assumption may not be exactly true for a small group of births, minor deviations from this assumption are unlikely to impact notably on estimates of median GA at birth. In particular, we observed that the strategy of treating pre‐eclamptic deliveries as a ‘competing risk’ in the survival analysis had little impact on the main estimates of median duration of pregnancy.

### Conclusions

We found close statistical agreement between ultrasound and ART‐based dating for both GA and EDD. However, ART‐based calculations of GA should use a median follicular‐phase duration of 15 days instead of 14 days to align the GA scale with that of LMP and ultrasound‐based methods. The birth distributions of ART and spontaneously conceived pregnancies differed. In particular, our estimates showed a median pregnancy duration of 286 days in frozen ET pregnancies, corresponding to a birth delay of approximately 3 days compared with pregnancies conceived spontaneously or via fresh ET. This difference was present regardless of the dating method used and was therefore largely independent of fetal ultrasound biometry in early pregnancy. As a consequence, ultrasound dating formulae derived from ART pregnancies may not be optimal for dating spontaneously conceived pregnancies. By the same token, clinical definitions of preterm and post‐term ART births should be re‐evaluated based on median pregnancy duration, considering updated risk estimates associated with fresh and frozen ET separately.

## Supporting information


**Appendix S1** Details regarding eSnurra (a population‐based ultrasound model).
**Appendix S2** Kaplan–Meier estimates of the cumulative birth distribution.
**Appendix S3** Comparison of pregnancy dating approaches.
**Appendix S4** Observed distribution of nonspontaneous onset of births.
**Appendix S5** Overview of study cohorts.
**Appendix S6** Sensitivity analyses.
**Figure S1** Histogram showing pairwise gestational age (GA) differences in days between the population‐based ultrasound model (GA_US_) and the assisted reproductive technology (ART)‐based formula using a median follicular‐phase duration of 15 days (GA_ART,15_) (calculated as GA_US_ − GA_ART,15_).
**Figure S2** Stacked bar charts displaying the observed birth distribution when using ultrasound‐based gestational age (GA_US_), stratified by nonspontaneous births (dark gray) and spontaneous births (light gray) in spontaneously conceived pregnancies (left panel), pregnancies conceived via fresh embryo transfer (center panel) and pregnancies conceived via frozen embryo transfer (right panel).
**Figure S3** Cumulative birth distribution (Kaplan–Meier) curves comparing pregnancies delivered after conception by fresh or frozen embryo transfer, using the extended dataset with all available data.
**Figure S4** Cumulative birth distribution (Kaplan–Meier) curves for pregnancies conceived after fresh intracytoplasmic sperm injection (ICSI), fresh *in‐vitro* fertilization (IVF), frozen ICSI or frozen IVF.
**Figure S5** Cumulative birth distribution curves constructed from censored quantile regression analyses, adjusted for maternal age at birth, parity and fetal sex.
**Figure S6** Cumulative birth distribution (Kaplan–Meier) curves comparing different strategies for handling nonspontaneous births.
**Table S1** Pregnancies conceived using assisted reproductive technology (ART) obtained from our main cohort, sampled from the Medical Birth Registry of Norway in 2015–2021, according to ART method and day of embryo transfer.
**Table S2** Pregnancies conceived using assisted reproductive technology (ART) obtained from the enlarged cohort, sampled from the Medical Birth Registry of Norway in 2015–2021, according to day of embryo transfer.

## Data Availability

The data that support the findings of this study are available from application through the portal for accessing such data in Norway: www.helsedata.no. Restrictions apply to the availability of these data, which were used under license for this study. Data are available after approvals and with the permission of Helsedataservice, available at https://helsedata.no/no/helsedataservice/.
