# Supplementary material for: Gestational age determination in pregnancies conceived via assisted reproductive technology
Source: Ultrasound Obstet Gynecol. 2026 Apr 30;67(5):604–12. doi: 10.1002/uog.70219 (PMC13136055; doi:10.1002/uog.70219)
Supplement: Supplementary file 1 — Appendix S1 Details regarding eSnurra (a population‐based ultrasound model). Appendix S2 Kaplan–Meier estimates of the cumulative birth distribution. Appendix S3 Comparison of pregnancy dating approaches. Appendix S4 Observed distribution of nonspontaneous onset of births. Appendix S5 Overview of study cohorts. Appendix S6 Sensitivity analyses. Figure S1 Histogram showing pairwise gestational age (GA) differences in days between the population‐based ultrasound model (GAUS) and the assisted reproductive technology (ART)‐based formula using a median follicular‐phase duration of 15 days (GAART,15) (calculated as GAUS − GAART,15). Figure S2 Stacked bar charts displaying the observed birth distribution when using ultrasound‐based gestational age (GAUS), stratified by nonspontaneous births (dark gray) and spontaneous births (light gray) in spontaneously conceived pregnancies (left panel), pregnancies conceived via fresh embryo transfer (center panel) and pregnancies conceived via frozen embryo transfer (right panel). Figure S3 Cumulative birth distribution (Kaplan–Meier) curves comparing pregnancies delivered after conception by fresh or frozen embryo transfer, using the extended dataset with all available data. Figure S4 Cumulative birth distribution (Kaplan–Meier) curves for pregnancies conceived after fresh intracytoplasmic sperm injection (ICSI), fresh in‐vitro fertilization (IVF), frozen ICSI or frozen IVF. Figure S5 Cumulative birth distribution curves constructed from censored quantile regression analyses, adjusted for maternal age at birth, parity and fetal sex. Figure S6 Cumulative birth distribution (Kaplan–Meier) curves comparing different strategies for handling nonspontaneous births. Table S1 Pregnancies conceived using assisted reproductive technology (ART) obtained from our main cohort, sampled from the Medical Birth Registry of Norway in 2015–2021, according to ART method and day of embryo transfer. Table S2 Pregnancies conceived using assisted reproductiv [file UOG-67-604-s001.pdf]

Supplementary appendix: Gestational age determination in pregnancies conceived via assisted reproductive technology

Miriam Gjerdevik[1,2], Hans Ivar Hanevik[2,3], Siri Eldevik Håberg[2,4], Håkon Kristian Gjessing[2,4]

1 Department of Computer science, Electrical engineering and Mathematical sciences, Western Norway University of Applied Sciences
2 Centre for Fertility and Health, Norwegian Institute of Public Health
3 Fertility Department, Telemark Hospital Trust
4 Department of Global Public Health and Primary Care, University of Bergen

Table of contents

Appendix S1: eSnurra—a population-based ultrasound model **3**

Appendix S2: Kaplan-Meier estimates of the cumulative birth distribution **3**

Appendix S3: A comparison of pregnancy dating approaches **3**

Appendix S4: The observed distribution of nonspontaneous onset of births **4**

Appendix S5: Overview of cohorts **6**

Appendix S6: Sensitivity analyses **7**
Selection bias 7
Intracytoplasmic sperm injection versus *in-vitro* fertilization 8
Adjusted survival models 9
Alternative strategies for analyses 10

Appendix S1: eSnurra—a population-based ultrasound model

The eSnurra ultrasound dating model was developed on a non-selected population from the geographically well-defined area of Trondheim, Norway, and its surrounding municipalities.[1] It has further been validated on several other Norwegian subpopulations.[2,3] While traditional sample-based ultrasound methods for pregnancy dating vary in predictive quality,[4] eSnurra performs more consistently and exhibits better precision. By proper calibration, it avoids selection bias and eliminates systematic error.[2,3] eSnurra is also recommended by the Norwegian Directorate of Health as the national tool for determining gestational age (GA) and estimated date of delivery (EDD) in Norway.[5] Ultrasound dating based on eSnurra (GA_{US}) was thus our method of choice for comparison with ART dating.

Appendix S2: Kaplan-Meier estimates of the cumulative birth distribution

Our strategy to differentiate spontaneous and nonspontaneous births is important when calculating the cumulative birth distribution $F(t)$. For instance, a substantial number of births are induced on indication of overdue pregnancy; that is, they are induced precisely because the pregnancy lasted longer than expected. Simply removing such pregnancies from the analysis would incorrectly bias the birth distribution downward. Similarly, treating non-spontaneous onset of births as spontaneous would create the impression that the biological age at birth is shorter than it really is. The effect of neglecting time-to-event analysis by these alternative approaches is demonstrated in our sensitivity analyses (Appendix S6, Figure S6).

To obtain estimates of the survival function $S(t)$ and the corresponding cumulative birth distribution $F(t)$, we calculated standard Kaplan-Meier curves. This strategy is based on the fact that for induced births, the spontaneous time of birth is not observed directly, but is known only to exceed the time of induction. If the requirement of "independent censoring" is satisfied, i.e., the censored pregnancies would have followed the birth distribution of spontaneous deliveries, then $F(t)$ represents the cumulative birth distribution as it would have been observed in a population where all births happened spontaneously. Note that when calculating median pregnancy duration from survival functions, it is not important whether inductions on postterm indication are treated as censored or spontaneous since they are well past the median. However, the time-to-event approach is particularly important for, for instance, elective sections prior to term, since it is unknown whether they would have passed term or not if they had proceeded to a spontaneous birth.

Appendix S3: A comparison of pregnancy dating approaches

Figure S1 displays the histogram of the pairwise differences between the eSnurra ultrasound model and the ART formula assuming a median follicular phase duration of 15 days, i.e., for each woman we calculated the individual difference $GA_{US} - GA_{ART,15}$. Fresh and frozen embryo transfers (ETs) were analyzed combined. For comparison, we superimposed two curves of smoothed frequencies: The pairwise differences in GA calculated by ultrasound and the last menstrual period (LMP) (solid black curve), and the pairwise differences in GA in late first-trimester dating using biparietal diameter (BPD) and crown-rump length (CRL) as the basis for the ultrasound calculations (dashed gray curve). The latter curve was extracted from another publication[6] (see below for details).

The median difference between GA_{US} and $GA_{ART,15}$ was -0.3 days (95% CI: -0.4 to -0.2). Counting differences in either direction, the difference was one day or less for 1792 of 4147 (43.2%) examinations, while 917 (22.1%) showed a difference of two days, 605 (14.6%) had a difference of three days, 375 (9.0%) had a difference of four days, and 458 (11.0%) exhibited a difference of five days or more.

Measured by GA_{US}, 268 of the 4147 ART births (6.5%, 95% CI: 5.8% to 7.3%) were classified as preterm (born on or before gestational day 258, i.e., prior to gestational week 37). In comparison, there were 294 preterm births using $GA_{ART,14}$ (7.1%, 95% CI: 6.3% to 7.9%), and 274 preterm births using $GA_{ART,15}$ (6.6%, 95% CI: 5.9% to 7.4%). In total, 60 pairs were discordant between GA_{US} and $GA_{ART,15}$, representing 1.4% of the ART births (95% CI: 1.1% to 1.9%). Measured both by GA_{US} and $GA_{ART,15}$, 28 births (0.68%, 95% CI: 0.47% to 0.97%) occurred on or before gestational day 223 (i.e., before gestational week 32), and 4 pairs were discordant (0.10%, 95% CI: 0.04% to 0.25%). In comparison, 31 children (0.75%, 95% CI: 0.53% to 1.06%) were born on or prior to gestational day 223 using $GA_{ART,14}$. There were no classification differences between the three dating methods with cut-off on gestational day 209 (i.e., born before gestational week 30), and 11 births were recorded by all three methods (0.27%, 95% CI: 0.15% to 0.47%). The binomial confidence intervals were obtained by the *binom.confint* function in the *binom* package (version 1.1-1.1) in R, using the Wilson method.

The differences in GA between ultrasound and LMP were based on 107,098 spontaneously conceived pregnancies from the Medical Birth Registry of Norway (MBRN) with a recorded LMP GA at birth $\leq$ 310 days. Each pregnancy was dated by eSnurra, and the reliability of the first day of LMP was recorded as certain. We observed a close symmetry around zero, which is as expected since the population-based ultrasound model is calibrated to have no systematic bias relative to LMP. The median difference was -0.12 days (95% CI: -0.14 to -0.09), demonstrating the accurate calibration of the ultrasound model even in a new population setting. The

absolute difference was one day or less for 34,526 (32.2%) of the measurements, while 18,200 (17.0%) exhibited an absolute difference of two days, 14,687 (13.7%) had a difference of three days, 11,015 (10.3%) had a difference of four days, and 28,670 (26.8%) had a difference of five days or more. The variance of the GA differences between ultrasound and LMP was clearly larger than that of the corresponding differences observed between ultrasound and ART, underscoring the close agreement between GA_{US} and $GA_{ART,15}$. Moreover, both ultrasound and ART dating eliminate the individual variation in follicular length. Our findings are also in line with previous results showing that EDD based on ultrasound exhibits a narrower birth distribution and outperforms LMP dating.[7]

The smoothed curve of GA differences between BPD and CRL was adapted from a recent study by Gjessing et al.[6] Their data were based on 11,041 pregnancies (with a total of 12,260 ultrasound examinations) from a non-selected population from a geographically delimited area surrounding Trondheim, Norway, sampled over the years from 1987 to 2017. Gjessing et al. found close consistency between BPD and CRL for the prediction of GA when incorporated into the same model for late first-trimester ultrasound dating. Specifically, the absolute difference in EDD between BPD and CRL was one day or less for 50.8% of their examinations, while 23.8% exhibited a difference of two days, 13.9% had a difference of three days, 6.5% had a difference of four days, and only 5.0% exhibited a difference of five days or more.[6] Thus, the precision between BPD and CRL was only marginally better than that observed between GA_{US} and $GA_{ART,15}$, again underscoring the close agreement between ultrasound and ART-based dating. Moreover, since BPD is as precise as CRL for late first-trimester ultrasound dating, and since there is less individual variability in fetal growth in early pregnancy, we expect population-based ultrasound predictions to perform even better as routine pregnancy dating in Norway is currently shifting from week 18 to week 12. However, since higher CRL values and divergent growth trajectories have been demonstrated at 6 to 14 weeks in frozen versus fresh cycles,[8] fetal growth discrepancies at the timing of ultrasound dating should be evaluated at the population level to mitigate GA bias and overestimation in frozen cycles.

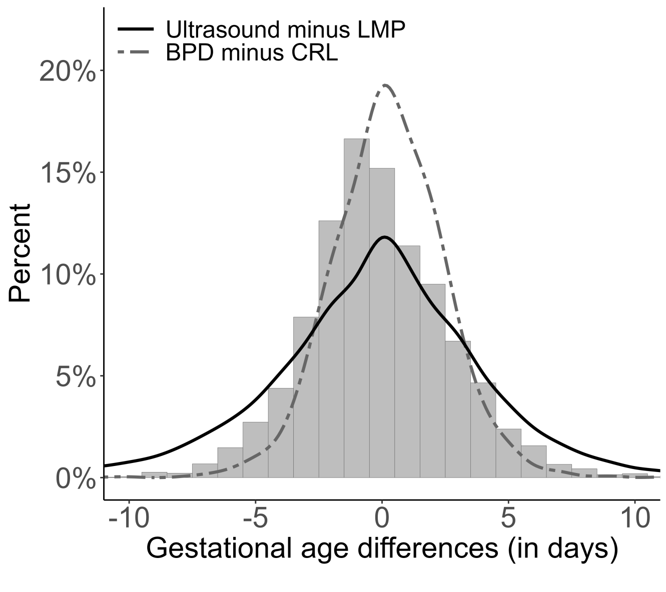

Figure S1: Histogram showing pairwise gestational age (GA) differences in days between the population-based ultrasound model (GA_{US}) and the assisted reproductive technology (ART)-based formula using a median follicular phase duration of 15 days ($GA_{ART,15}$) (calculated as $GA_{US} - GA_{ART,15}$). Fresh and frozen cycles were combined. The solid black curve shows the smoothed GA differences as calculated by GA_{US} minus last menstrual period (LMP) in spontaneously conceived pregnancies. The dashed gray curve shows the smoothed GA differences between ultrasound biparietal diameter (BPD) and ultrasound crown-rump length (CRL), as adapted by Gjessing et al.[6] Outliers are not plotted.

Appendix S4: The observed distribution of nonspontaneous onset of births

To assess the extent of nonspontaneous onset of births among spontaneously conceived, fresh, and frozen ETs, we constructed stacked bar charts of the observed birth distribution over GA_{US} (Figure S2). The relative distribution of nonspontaneous onset of deliveries is shown in dark gray, and the relative distribution of spontaneous onset of deliveries is shown in light gray.

The onset of birth was recorded as spontaneous for 113,379 of 163,544 spontaneously conceived children (69.3%), 1333 of 2067 children conceived after fresh ET (64.5%), and 1162 of 2080 children conceived after frozen ET (55.9%). Consequently, the remaining figures for nonspontaneous onset of births were 50,165 (30.7%)

for spontaneously conceived children, 734 (35.5%) for fresh ETs, and 918 (44.1%) for frozen ETs.

For all three groups, the distribution of nonspontaneous births can be described as trimodal, with peaks at approximately 273–274 days (1.5 weeks before term), 283–286 days (due date and immediately after), and 294–295 days (11–12 days after due date). The first peak corresponds to the first round of elective births (by cesarean section or induction), including, among others, women with known diabetes who should be considered for induction from gestational week 38.[9] The second peak likely represents inductions without medical or obstetric indications, often referred to as inductions on maternal request or elective inductions, i.e., in the absence of medical or obstetrical factors. This group might represent multiparous mothers with previous traumatic birth experiences.[10] The last peak corresponds to the Norwegian guidelines concerning postterm guidelines,[11] which state that births must be induced no later than 11 days past due date. Thus, on gestational days 294 and later, the majority of births were induced, and differences in the frequency of nonspontaneous onset of births stem from shifts in the observed birth distributions. Consequently, the higher frequency of nonspontaneous onset of postterm births in frozen cycles results from the prolonged pregnancy duration. One can also observe a "shoulder" of an increasing proportion of inductions around 260–265 days of GA, which corresponds to the approximate limit of term pregnancies. This proportion of induced births may include risk pregnancies with observed growth abnormalities or preeclampsia, where one balances the risk of continuing versus terminating the pregnancy. While the differences in distributions between spontaneously conceived children and fresh ETs may partly be attributed to unequal sample sizes, there was an overall larger proportion of nonspontaneous births among frozen ETs.

We note that the histograms in Figure S2 describe the observed and actual birth distribution in our population and contribute useful information on clinical practices and guidelines, as well as potential social trends. However, they provide biased estimates of the biological timing of birth, since the high number of nonspontaneous onset of births shifts the GA to lower values.

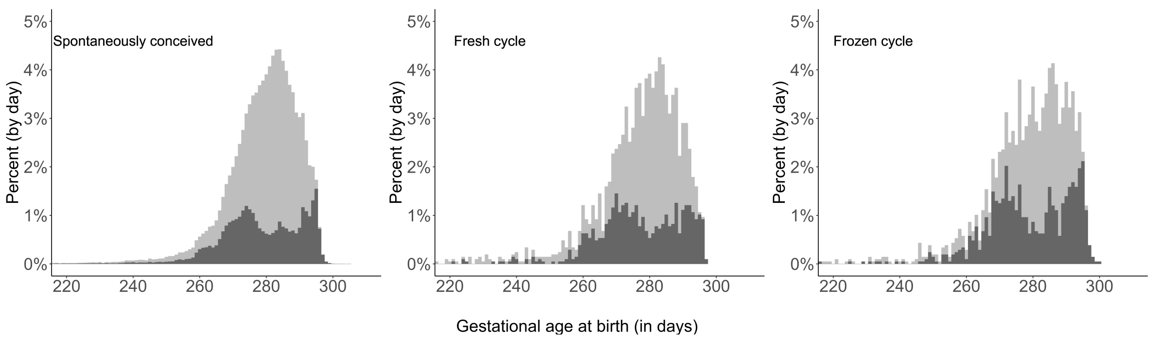

Figure S2: Stacked bar charts displaying the observed birth distribution over ultrasound-based gestational age (GA_{US}), stratified by nonspontaneous births (dark gray) and spontaneous births (light gray) in spontaneously conceived pregnancies (left panel), pregnancies conceived via fresh embryo transfer (center panel), and pregnancies conceived via frozen embryo transfer (right panel).

Appendix S5: Overview of cohorts

Our main cohort was established from MBRN in 2015–2021. It included 163,544 spontaneously conceived children, 2067 children conceived after fresh ET, and 2080 children conceived after frozen ET, as described in *Methods* and *Results* of the main article. The ART samples were further categorized into *in-vitro* fertilization (IVF) and intracytoplasmic sperm injection (ICSI), and day of ET, as showcased in Table S1. The main cohort was used for the analyses in figures 1, 2, and 3a of the main article and figures S1 (histogram), S2, S4, S5, and S6 of the supplementary appendix.

Increased sample sizes were obtained by including all ART pregnancies, i.e., without restricting our dataset to pregnancies specifically known to be dated by eSnurra. This extended ART sample underwent the same inclusion and exclusion criteria as the main cohort (see *Methods* and *Results*) and comprised a total of 8945 children conceived with fresh ET and 4795 children conceived with frozen ET. The cohort was further categorized into day of ET, and the numbers are shown in Table S2. This enlarged dataset was used for the analyses in Figure 3b of the main article and Figure S3 of the supplementary appendix. We note that the large increase in ART pregnancies that met the criteria of this study is primarily attributed to the EDD being determined based on the known dates of oocyte retrieval and ET rather than ultrasound.

Table S1: Pregnancies conceived using assisted reproductive technology (ART) obtained from our main cohort, sampled from the Medical Birth Registry of Norway in 2015–2021, according to ART method and day of embryo transfer.

	Fresh cycle (N = 2067)	Frozen cycle (N = 2080)
IVF	1126	1263
ICSI	941	815
2-day transfer	922	245
3-day transfer	500	117
5-day transfer	569	1630

We were unable to categorize two frozen cycles as either IVF or ICSI.
1-, 4-, and 6-day transfers were not analyzed separately due to limited sample sizes. IVF: *in-vitro* fertilization; ICSI: intracytoplasmic sperm injection.

Table S2: Pregnancies conceived using assisted reproductive technology (ART) obtained from the enlarged cohort, sampled from the Medical Birth Registry of Norway in 2015–2021, according to day of embryo transfer.

	Fresh cycle (N = 8945)	Frozen cycle (N = 4795)
2-day transfer	4863	1137
3-day transfer	1904	537
5-day transfer	1856	2868

1-, 4-, and 6-day transfers were not analyzed separately due to limited sample sizes.

Appendix S6: Sensitivity analyses

Selection bias

In the main analyses, we omitted observations for which the population-based ultrasound model eSnurra was not employed in the estimation of GA. To examine the effect of this exclusion, we performed sensitivity analyses that included these observations. Thus, in Figure S3, we calculated the cumulative birth distributions for fresh and frozen ETs using $GA_{ART,15}$, employing all the available data from MBRN in 2015–2021. Without restricting to eSnurra samples, our cohort included 8945 fresh cycles and 4795 frozen cycles from MBRN in 2015–2021 (see Appendix S5). The median $GA_{ART,15}$ at birth was 283.2 days (95% CI: 283.0 to 283.4) in fresh cycles and 286.1 days (95% CI: 285.7 to 286.6) in frozen cycles, corresponding to a small upward shift of less than half a day compared to the restricted sample (see Figure 2 of the main article). The difference in the median $GA_{ART,15}$ between frozen and fresh ETs was 2.9 days (95% CI: 2.4 to 3.4) in the extended dataset, which is consistent with the difference observed in Figure 2 of the main article.

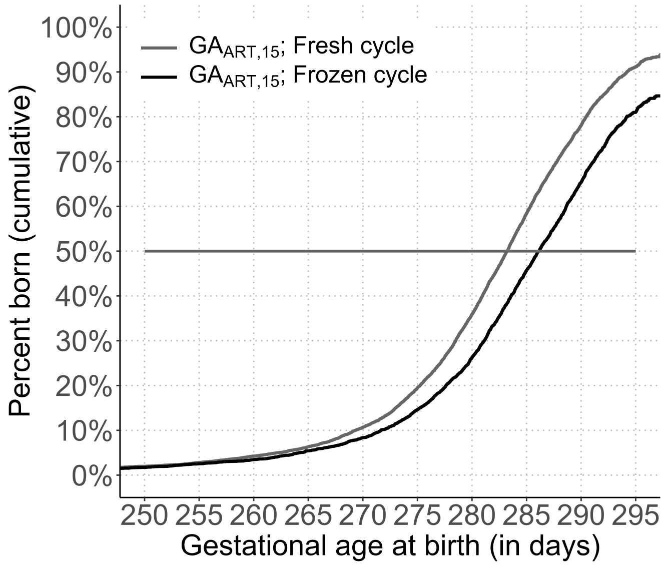

Figure S3: Cumulative birth distribution (Kaplan-Meier) curves comparing children born after fresh and frozen embryo transfer using the extended dataset with all available data. A time-to-event approach was used, treating nonspontaneous onset of births as censored observations. The gestational age at birth was based on the assisted reproductive technology (ART) formula with a median follicular phase duration of 15 days ($GA_{ART,15}$). Both the event and censoring times were recorded on the ART-derived timescale.

Intracytoplasmic sperm injection versus *in-vitro* fertilization

Figure S4 illustrates the cumulative birth distributions for fresh and frozen ETs categorized by the fertilization method, either ICSI or IVF. Among children conceived after fresh ET, our sample included 941 children fertilized by ICSI and 1126 by IVF. Among children conceived after frozen ET, 815 children were fertilized by ICSI and 1263 were fertilized by IVF (see Appendix S5).

For children fertilized by ICSI, the median GA_{US} at birth was 283.1 days (95% CI: 282.4 to 284.2) in fresh cycles and 285.8 days (95% CI: 284.8 to 286.8) in frozen cycles, which constitutes a difference in median GA_{US} of 2.7 days (95% CI: 1.1 to 3.9) between frozen and fresh cycles. For children fertilized by IVF, the median GA_{US} at birth was 282.7 days (95% CI: 282.0 to 283.3) in fresh cycles and 286.2 days (95% CI: 285.5 to 287.0) in frozen cycles, a difference between frozen and fresh cycles of 3.6 days (95% CI: 2.6 to 4.5). For both fresh and frozen ETs, the curves were close to each other, indicating small differences in GA_{US} between ICSI and IVF. For fresh ETs, the difference in median GA_{US} between ICSI and IVF was 0.4 days (95% CI: −0.5 to 1.7). For frozen ETs, the difference in median GA_{US} between ICSI and IVF was −0.4 days (95% CI: −1.8 to 0.8).

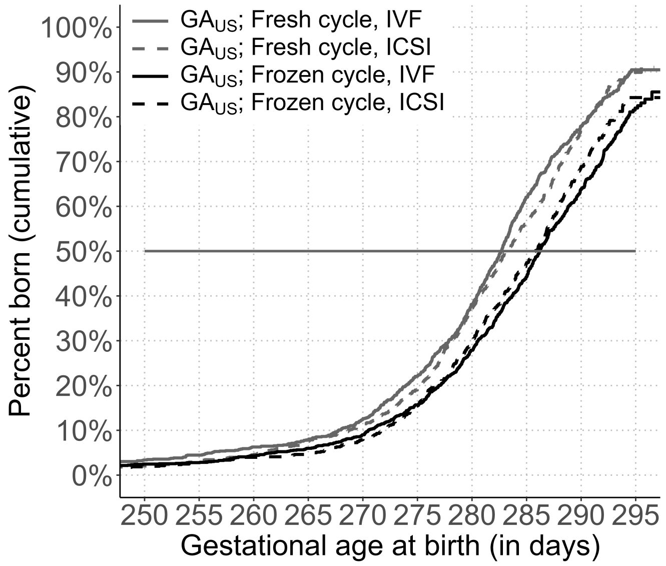

Figure S4: Cumulative birth distribution (Kaplan-Meier) curves for pregnancies conceived after fresh intracytoplasmic sperm injection (ICSI), fresh *in-vitro* fertilization (IVF), frozen ICSI, and frozen IVF. We applied a time-to-event approach and treated nonspontaneous onset of births as censored observations. The gestational age at birth was calculated using ultrasound (GA_{US}).

Adjusted survival models

Potential covariates may affect the timing of birth and residual variability. Thus, we performed censored quantile regression[12] and adjusted for maternal age at birth (age $<$ 30 years (45.9%), 30 years $\leq$ age $<$ 35 years (35.6%), age $\geq$ 35 years (18.4%)), parity (39.6% nulliparous and 60.4% parous), and child's sex (51.1% boys and 48.9% girls). The results were obtained using the *crq* function in the *quantreg* package (version 5.94) in R, employing the estimation method of Peng and Huang.[13] We then predicted cumulative birth curves for the "average" pregnancy conceived spontaneously or in fresh or frozen cycles, using the population proportions of each covariate via dummy variables for the non-reference categories. The results are displayed in Figure S5. The predicted median GA_{US} at birth was 283.5 days for the "average" spontaneously conceived pregnancy, 282.7 days for fresh cycles, and 285.8 days for frozen cycles. These estimates align closely with those of the unadjusted analyses (Figure 2 of the main article).

Although our overall findings provide strong evidence that ART pregnancies are different from those conceived spontaneously, we were unable to identify and adjust for all relevant covariates that can influence the timing of birth. In particular, potential parental selection mechanisms into fresh or frozen ETs should be further explored. Nonetheless, our results imply that the prolonged duration of pregnancy in frozen cycles relates to a later onset of labor and not an initial "hibernation" period following ET, which may guide future research.

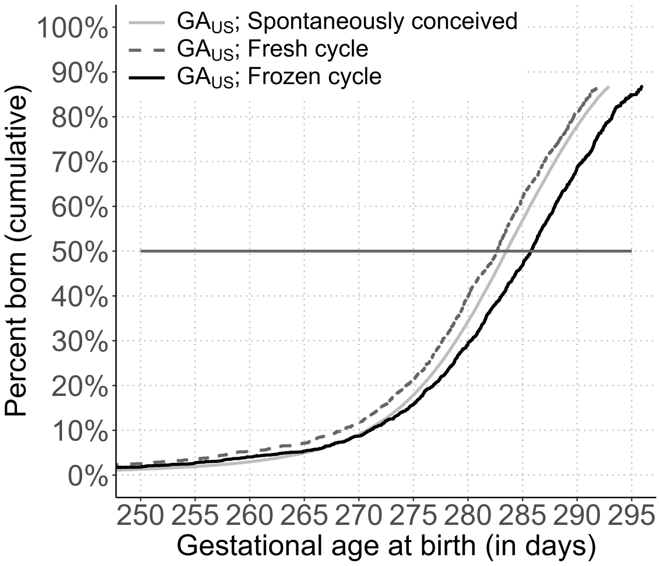

Figure S5: Cumulative birth distribution curves constructed from censored quantile regression analyses, adjusted for maternal age at birth, parity, and fetal sex. We used the mean value of each covariate to predict curves corresponding to an "average" pregnancy. The gestational age at birth was calculated using ultrasound (GA_{US}).

Alternative strategies for analyses

In Figure S6, we compared the cumulative GA distributions of three alternative approaches for handling non-spontaneous onset of deliveries: 1) Constructing regular Kaplan-Meier curves in a time-to-event framework, i.e., the optimal strategy; 2) Treating nonspontaneous deliveries as if the onset of birth happened spontaneously, i.e., as events without censoring; 3) Simply excluding nonspontaneous births from the cohort. Results are shown for frozen ETs only. As expected, not distinguishing between spontaneous and nonspontaneous deliveries shifted the distribution toward lower values, with a median GA at birth of 280.8 days (95% CI 280.3 to 281.6). The strategy of excluding nonspontaneous births also resulted in lower values and a median GA at birth of 281.5 days (95% CI 280.8 to 282.2). The considerable negative bias induced by alternatives 2) and 3) strongly highlights the need for a time-to-event approach for correct assessment of bias and precision in EDD.

Since preeclampsia is a common medical indication of iatrogenic delivery, more detailed analyses might be achieved by treating preeclamptic pregnancies as a separate category. It is often recommended to do this by assuming that preeclamptic births act as a "competing risk" to non-preeclamptic births.[14] In our data we did not have specific information on indication for inductions or cesarean sections. We did, however, have information on whether the mother had preeclampsia. We thus performed a survival analysis where nonspontaneous non-preeclamptic births were treated as censored, all preeclamptic births were treated as one event type, and spontaneous non-preeclamptic births were treated as a second event type. We then estimated the cumulative GA distribution for the second event type as a cumulative incidence function.[15] Note that a cumulative incidence function falls below the standard cumulative distribution curve. As seen from Figure S6, the difference around median time between the standard survival curve and the cumulative incidence curve was minimal. The median time at birth using the competing risk strategy was 286.7 days (95% CI 286.1 to 287.1), although it should be remarked that a median time is harder to interpret in a cumulative incidence curve.

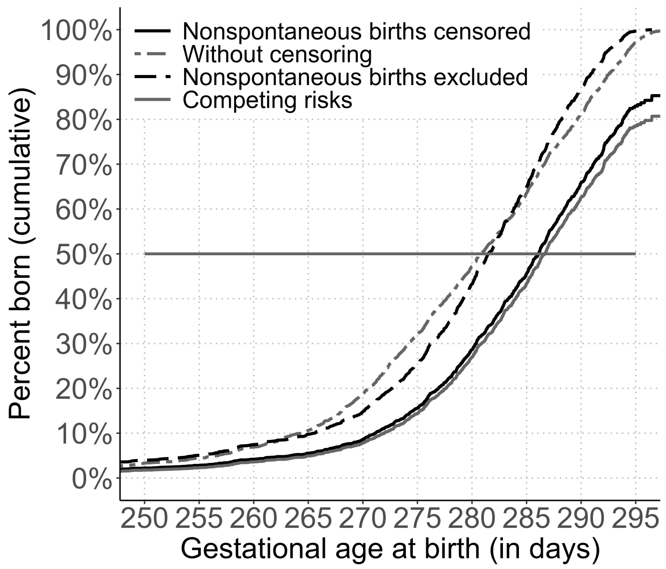

Figure S6: Cumulative birth distribution (Kaplan-Meier) curves comparing different strategies for handling nonspontaneous births. *Nonspontaneous births censored* treats nonspontaneous onset of births as censored observations and is the preferred option. *Without censoring* treats nonspontaneous and spontaneous onset of births equally. *Nonspontaneous births excluded* removes nonspontaneous onset of births from the cohort. *Competing risks* treats preeclampsia as a competing event. The gestational age at birth was calculated using ultrasound (GA_{US}). Results are shown for frozen embryo transfers.

References

[1] Gjessing HK, Grøttum P, Eik-Nes SH. A direct method for ultrasound prediction of day of delivery: a new, population-based approach. *Ultrasound Obstet Gynecol*. 2007;30(1):19-27.

[2] Økland I, Gjessing HK, Grøttum P, Eggebø TM, Eik-Nes SH. A new population-based term prediction model vs. two traditional sample-based models: validation on 9046 ultrasound examinations. *Ultrasound Obstet Gynecol*. 2011;37(2):207-13.

[3] Økland I, Nakling J, Gjessing HK, Grøttum P, Eik-Nes SH. Advantages of the population-based approach to pregnancy dating: results from 23,020 ultrasound examinations. *Ultrasound Obstet Gynecol*. 2012;39(5):563-8.

[4] Økland I, Gjessing HK, Grøttum P, Eik-Nes SH. Biases of traditional term prediction models: results from different sample-based models evaluated on 41 343 ultrasound examinations. *Ultrasound Obstet Gynecol*. 2010;36(6):728-34.

[5] The Norwegian Directorate of Health. Brev fra Helsedirektoratet til regionale foretak, ref 12/6746-24. Oslo; 2014.

[6] Gjessing HK, Grøttum P, Dreier JM, Eik-Nes SH. Biparietal diameter vs crown-rump length as standard parameter for late first-trimester pregnancy dating. *Ultrasound Obstet Gynecol*. 2024;64(6):739-45.

[7] Tunón K, Eik-Nes SH, Grøttum P. A comparison between ultrasound and a reliable last menstrual period as predictors of the day of delivery in 15,000 examinations. *Ultrasound Obstet Gynecol*. 1996;8(3):178-85.

[8] Cavoretto PI, Farina A, Girardelli, et al. Greater fetal crown-rump length growth with the use of in vitro fertilization or intracytoplasmic sperm injection conceptions after thawed versus fresh blastocyst transfers: secondary analysis of a prospective cohort study. Fertil Steril. 2021;116(1):147-56.

[9] The Norwegian Directorate of Health. Nasjonal faglig retningslinje for diabetes (online document). Oslo; 2016. Latest professional change 2019-12-20, accessed 2025-05-14. `https://www.helsedirektoratet.no/retningslinjer/diabetes/svangerskap-ved-kjent-diabetes#igangsetting-av-fodsel-og-glukosekontroll-under-og-etter-fodsel-hos-gravide-med-kjent-diabetes`.

[10] Dögl M, Romundstad P, Berntzen, et al. Elective induction of labor: a prospective observational study. *PLoS One*. 2018;13(11):e0208098.

[11] The Norwegian Directorate of Health. Gravide som har passert termin bør senest igangsettes for fødsel uke 42+0 (online document). Oslo; 2024. Latest professional change 2024-03-04, accessed 2025-05-14. `https://www.helsedirektoratet.no/retningslinjer/fodselsomsorgen/svangerskap-passert-termin/gravide-som-har-passert-termin-bor-senest-igangsettes-for-fodsel-uke-42-0`.

[12] Koenker R. Censored quantile regression redux. J Statistical Software. 2008;27(6):1-25.

[13] Peng L, Huang Y. Survival analysis with quantile regression models. J Am Stat Assoc. 2008;103(482):637-49.

[14] Wright D, Wright A, Nicolaides KH. The competing risk approach for prediction of preeclampsia. Am J Obstet Gynecol. 2020;223(1):12-23.e7.

[15] Aalen OO, Borgan Ø, Gjessing HK. Survival and event history analysis: a process point of view. New York, NY: Springer Verlag; 2008.
